# Comprehensive genome analysis of *Mycobacterium avium* subsp. *paratuberculosis* in camels from Saudi Arabia: Molecular epidemiology and antimicrobial resistance

**DOI:** 10.14202/vetworld.2025.859-876

**Published:** 2025-04-19

**Authors:** M. Salem, A. Zaghawa, F. Housawi, Ahmed Elsify, Mohamed E. Hasan, Alyaa Elrashedy, Kazem AlMohammed Salem, Nidhal Ben Amor, A. Al Naeem

**Affiliations:** 1 Department of Clinical Sciences, College of Veterinary Medicine, King Faisal University, Hofuf, Kingdom of Saudi Arabia; 2 Department of Medicine and Infectious Diseases, Faculty of Veterinary Medicine, Cairo University, Giza, Egypt; 3 Department of Animal Medicine and Infectious Diseases, Faculty of Veterinary Medicine, University of Sadat City, Sadat City, Minoufiya, Egypt; 4 Department of Bioinformatics, Genetic Engineering and Biotechnology Research Institute, University of Sadat City, Sadat City, Egypt; 5 Department of Health Information Technology, Faculty of Health Science Technology, Borg Al Arab Technological University (BATU), Alexandria, Egypt; 6 Animal Resources Management, Ministry of Environment, Water and Agriculture, Al-Ahsa, Saudi Arabia; 7 Department of Public Health, College of Veterinary Medicine, King Faisal University, Hofuf, Kingdom of Saudi Arabia

**Keywords:** antimicrobial resistance, camel health, *Mycobacterium avium* subsp. *paratuberculosis*, phylogenetics, Saudi Arabia, whole-genome sequencing

## Abstract

**Background and Aim:**

Paratuberculosis, caused by *Mycobacterium avium* subsp. *paratuberculosis* (MAP), is a significant infectious disease affecting livestock, including camels in Saudi Arabia, leading to severe economic losses. Despite its impact, limited genomic studies have been conducted to characterize MAP strains in camels and their antimicrobial resistance (AMR) profiles. This study aimed to (1) characterize the clinical and pathological findings of MAP infections in camels; (2) determine the seroprevalence of MAP in the eastern region of Saudi Arabia; (3) differentiate between MAP strains using polymerase chain reaction (PCR) and bioinformatics tools; (4) conduct a comprehensive genomic analysis; and (5) identify genes associated with AMR, virulence, and immune response.

**Materials and Methods:**

A total of 345 blood samples were collected for seroprevalence analysis using enzyme-linked immunosorbent assay (ELISA), and 68 rectal scraping samples were analyzed using Ziehl–Neelsen staining and PCR for strain differentiation. Seventeen clinical cases underwent clinical, postmortem, and histopathological examinations. Whole-genome sequencing and bioinformatics analyses were performed using the Bacterial and Viral Bioinformatics Resource Center to identify genetic variations, *AMR* genes, and phylogenetic relationships among MAP isolates.

**Results:**

Clinical findings revealed progressive emaciation and chronic diarrhea in affected camels. Gross examination showed intestinal wall thickening and mesenteric lymph node congestion. Histopathological analysis indicated hyperactivation of crypts of Lieberkühn and mononuclear cell infiltration. PCR analysis identified a higher prevalence of the sheep (S) strain (162 bp) compared to the cattle (C) strain (310 bp). The overall seroprevalence of MAP was 8.11% (ELISA). Whole-genome sequencing identified 34 *AMR* genes and 10 virulence genes, with annotation revealing 4.7 million base pairs, coding sequences, transfer RNA, ribosomal RNA, and pseudogenes. Phylogenetic analysis grouped MAP strains into four distinct clades, indicating potential cross-species transmission.

**Conclusion:**

This study provides critical insights into the genetic diversity and AMR mechanisms of MAP strains in camels, emphasizing the need for targeted control strategies. The findings highlight potential zoonotic risks and inform future vaccine development to mitigate MAP infections in livestock.

## INTRODUCTION

Camels serve multiple purposes, providing essential resources such as meat, milk, and wool. In addition, they are widely used for transportation and agricultural work, particularly in desert regions [1]. However, camel production in Saudi Arabia faces several challenges, including drought-induced pasture loss and the threat of infectious diseases. Among these, Johne’s disease (JD) is a major concern affecting camel populations in the Kingdom of Saudi Arabia. JD, caused by *Mycobacterium avium* subsp. *paratuberculosis* (MAP), is a chronic enteric infectious disease that results in substantial economic losses due to reduced milk production, infertility, and mortality. Furthermore, MAP has zoonotic significance, as it can be transmitted to humans through contaminated milk, potentially contributing to Crohn’s disease [2].

MAP is primarily transmitted through the fecal-oral route, where contamination of teats, milk, or colostrum facilitates direct transmission from infected to susceptible animals [3, 4]. Additional sources of infection include environmental contamination and the introduction of infected animals into previously uninfected herds [5–9]. Once established within a herd, JD progresses through four distinct stages, each characterized by varying clinical symptoms and diagnostic opportunities [10]. Genetic studies have identified variations in MAP strains based on *IS900* [11], *IS1311* [12], and *gyrB* sequences [13], revealing two primary strain types with host-specific preferences: Type II (cattle strain, MAP-C) and Type I/III (sheep strain, MAP-S) [14]. The sheep strain (MAP-S) predominantly infects sheep, has a slow growth rate, and exhibits yellow-orange pigmented colonies [15]. In contrast, the cattle strain (MAP-C) infects a broader range of hosts, grows more rapidly, and typically produces non-pigmented colonies [11]. While these strain characteristics have been well-documented, the genetic basis underlying host preference remains largely unknown.

Although JD has been extensively studied in cattle, research on its occurrence and genetic diversity in camels remains limited. The challenges in isolating MAP from diseased camels have hindered a comprehensive understanding of its epidemiology. This limitation is particularly evident in exotic animal species, where the ability to separate causal pathogens from clinical cases remains a significant challenge. A study by Alhebabi and Alluwaimi [16] reported that enzyme-linked immunosorbent assay (ELISA) and polymerase chain reaction (PCR) are reliable diagnostic tools for detecting JD in camels. However, further research is needed to evaluate their effectiveness in longitudinal disease control programs for Saudi camel herds. While PCR is often preferred over ELISA due to its practicality in MAP detection, its predictive value remains low due to variations in bacterial shedding patterns. Therefore, PCR is most effective when combined with ELISA for detecting MAP in milk or serum. Salem *et al*. [17] found that, despite certain limitations, bacterial culture remains the gold standard for MAP diagnosis. Molecular diagnostic tools, particularly PCR, can improve the detection of true-positive and true-negative cases, but primer specificity and sensitivity require careful consideration.

Previous studies by Sweeney *et al*. [3], Alhebabi and Alluwaimi [16], and Salem *et al*. [17] have successfully cultivated MAP from multiple infected camel samples exhibiting intermittent, chronic diarrhea indicative of JD. A significant proportion of these isolates were identified as belonging to the MAP-S strain through gene-based typing, suggesting possible transmission from infected sheep populations. Further epidemiological screening revealed that while MAP-S strains circulated within camel herds associated with sheep, no evidence of transmission was observed from goats [18]. Genomic analysis of camel isolates confirmed their classification as a sub-lineage of the sheep strain, indicating either host adaptation or regional genetic variations [19].

MAP strains are categorized into two major groups: Type C (cattle-type) and Type S (sheep-type) [20]. Recent research has identified cattle-type strains capable of cross-species transmission, demonstrating no strict host preference [20]. Several factors complicate JD control, including the disease’s prolonged incubation period, the bacterium’s resistance to antibiotics, and the limited sensitivity of existing diagnostic tests. However, implementing appropriate management strategies can significantly reduce disease prevalence within herds [21].

Given Saudi Arabia’s geographical position bordering Qatar, Kuwait, Iraq, Oman, and the United Arab Emirates, the eastern region serves as a primary entry point for various pathogens. Despite this, no vaccination program for JD in camels currently exists. Vaccination remains the most effective strategy for controlling infectious diseases. Identifying the circulating MAP strain in camel populations is essential for designing an effective vaccination program and assessing its potential protective efficacy [22].

Despite the widespread impact of MAP on livestock, including camels, limited studies have explored its genetic diversity, epidemiology, and antimicrobial resistance (AMR) in camel populations, particularly in Saudi Arabia. Most existing research focuses on MAP infections in cattle and sheep, leaving a significant knowledge gap regarding the strain variations, host adaptability, and cross-species transmission in camels. In addition, the genetic basis of MAP virulence, AMR mechanisms, and its potential zoonotic implications in camels remain largely unexamined. The current diagnostic tools, such as ELISA and PCR, exhibit limitations in predictive accuracy, necessitating further investigation into more effective detection and control strategies. Furthermore, no vaccination program currently exists for JD in camels, highlighting the need for genomic insights to inform vaccine development and disease management strategies.

This study aims to comprehensively investigate MAP infections in camels from the Eastern region of Saudi Arabia by integrating clinical, pathological, serological, and molecular analyses. Specifically, the study seeks to: (1) Determine the seroprevalence of MAP in camels using ELISA. (2) Differentiate between MAP strains through PCR-based molecular identification. (3) Conduct whole-genome sequencing and bioinformatics analysis to characterize the genetic composition of MAP isolates. (4) Identify *AMR* genes, virulence factors, and immune-related genes associated with MAP infections. (5) Assess phylogenetic relationships among camel-derived MAP strains and compare them with strains from other host species to evaluate cross-species transmission potential.

## MATERIALS AND METHODS

### Ethical approval

This study was approved by the Institutional Animal Ethics Committee of the College of Veterinary Medicine, King Faisal University, Al-Hasa, Kingdom of Saudi Arabia (KFU-REC-2023-OCT-ETHICS1516).

### Study period and location

This study was conducted from April 2021 to March 2023 at Eastern region of Saudi Arabia (Riyadh and Alhassa). The samples were processed at Laboratory of Department of Clinical Sciences, College of Veterinary Medicine, King Faisal University, Hofuf, Kingdom of Saudi Arabia.

### Sample collection and serological examination

Table 1 illustrates the number of camels and types of samples for each region under study. A total of 345 blood samples were collected from the jugular vein in vacutainer tubes for serum collection for the detection of antibodies against MAP using ELISA according to the manufacturer’s instructions, IDEXX^®^ ELISA kit (IDEXX, Netherlands). Among them, 17 diseased camels were examined thoroughly for a concise description of the symptoms and 3 camels from the diseased camels were slaughtered for a description of postmortem findings according to Peter and Cockcroft [23] and Griffiths [24] and samples collected from (intestine, liver, spleen, and mesenteric lymph node) for histopathological examination according to Bancroft and Gamble [25].

**Table 1:** Sample types, numbers, and locations.

Location	Animals		Samples	
	
	Apparently healthy	Diseased	Blood	Rectal scraping with feces
Al Zaydiyah	44	3	47	8
Haradh	36	1	37	7
Alsaih	8	0	8	1
Salwa	29	2	31	11
Hofuf	15	1	16	7
Tamani	6	2	8	2
Al Udayd	8	0	8	4
Fadillah	10	0	10	1
Ahsa Market	35	1	36	10
alghwaibah	20	0	20	3
Udhailiyah	20	2	22	3
Assarrar	75	4	79	1
Hanidh	10	0	10	1
Elwanan	12	1	13	9
Total	328	17	345	68

### Microscopy and molecular detection of MAP

Sixty-eight rectal scraping samples mixed with fecal matter were collected for MAP detection using Ziehl–Nelson stain, in which samples were deemed positive if two or more groups of three or more acid–fast Mycobacteria were found [26]. DNA extraction from the rectal scraping was performed as previously described by Clark *et al*. [27]. The extracted DNA was purified from the supernatant using QIAamp^®^ PowerFecal^®^ kit (QIAGEN, Courtaboeuf, France) according to the manufacturer’s instructions, and the purified DNA was stored at a frozen temperature. To distinguish between sheep (S-type) and cattle (C-type), three distinct PCRs were performed using template DNA from fecal samples. To identify the MAP insertion sequence IS900, the purified DNA was amplified. To distinguish between sheep (S-type) and cattle (C-type), a particular digital microfluidic chip PCR (DMC-PCR) was carried out. For the C-type, the estimated amplicon size would be 310 bp, whereas for the S-type, it would be 162 bp. Sheep MAP type-I-specific representational difference analysis (RDA) segments (pig-RDA10, pig-RDA20, and pig-RDA30) were amplified. Two microliters of each pure genomic DNA were amplified using the HotStartTaq^®^ plus master mix kit (QIAGEN, Germantown, MD, USA), and an I cycler PCR machine (Bio-Rad, Hercules, CA, USA) was used to perform the PCR procedures. Table 2 lists the primers, annealing temperature, and anticipated size of the DNA product for each PCR condition [28].

**Table 2:** Primers, annealing temperatures, and product sizes used in this study.

Primer name	Primer sequences	Annealing (°C)	Product size (bp)	Reference
IS900-F	5 CCTTTCTTGAAGGGTGTTCG 3	58	548	[28]
IS900-R	5 CCACCAGATCGGAACGTC 3			
DMC1-529 F	5 GCTGTTGGCTGCGTCATGAAG 3	60	310 (C-type)	
DMC1-531 F	5 TCTTATCGGACTTCTTCT GGC 3			
DMC1-533 R	5 CGGATTGACCTGCGTTTCAC, 3			162 (S-type)
pig-RDA10 P19	5 TAG CGG TCC CGC AGT TTG GC 3	61	382	
pig-RDA10 P20	5 TCA AGC CGA ACG AGG TGG TCG 3		382	
pig-RDA20 P21	5 TCG TCC CGT CCC GAT GCT GT 3		525	
pig-RDA20 P22	5 TGA GTC CTG TCG TGC ATG CG 3			
pig-RDA30 P23	5 TGA AGA GCC CGG ACA AGG GG 3			
pig-RDA30 P24	50 TAG GTC TCA GTG GTC CAC CAG C 3			

RDA=Representational difference analysis, DMC=Digital microfluidic chip

### Genome data acquisition and assembly

To conduct the bioinformatic study on MAP, we obtained information about MAP in Saudi Arabia from the National Center for Biotechnology Information’s bioproject database using accession number PRJNA68575 [18]; afterward, comprehensive genome analysis was performed using the meta-service on the server of the Bacterial and Viral Bioinformatics Resource Center (BV-BRC), which obtains raw data or single or paired reads to compute genome assembly, annotation, quality control, AMR, and candidate genes specific for crucial roles [29]. For genome assembly, the Unicycler (v0.4.8) pipeline was utilized, (https://github.com/rrwick/Unicycler), specifically applying the “AUTO” selection to assemble paired-end reads into short-read contig. Unicycler integrates the SPAdes (St. Petersburg genome assembler) *de novo* assembler to construct an initial assembly graph, which is then enhanced by leveraging both short and long reads (http://cab.spbu.ru/software/spades/). This process improves sequence continuity, resolves complex repeat regions, and generates high-quality circularized genome assemblies [29].

### Phylogenetic and comparative genomic analyses

Using MAUVE, multiple sequence alignment (MSA) was performed [30]. The reference genome, S397 (s-type III) of MAP was used. To provide an accurate picture, we also employed a BV-BRC phylogenetic tree known as “Codon Trees” to determine the evolutionary relationship between our strains and other outgroup strains. The pre-established PATRIC protein global families (PGFams) were used to select between 10 and 1000 single-copy families from among members of a given genomic group. The muscle is used to create alignments for protein sequences in every family [31]; and for their corresponding nucleotide sequences, BioPython’s codon-align function was employed [32]. In RaxML (Randomized Axelerated Maximum Likelihood), a phylogenetic inference software developed by Alexandros Stamatakis and maintained by the Heidelberg Institute for Theoretical Studies (HITS), 100 rapid bootstrap rounds were performed to generate confidence values. RAxML utilizes the maximum likelihood (ML) algorithm with rapid bootstrap resampling to estimate phylogenetic tree confidence values [33].

### Variant detection and cross-species analysis

The variation analysis tool on the BV-BRC server identifies genomic differences in clade 2 among sheep, camels, and pigs to investigate cross-species transmission. Using MAP JQ5 as the reference genome (where JQ refers to the strain name), the tool detects single nucleotide variants (SNVs) and short insertions/deletions (indels) from aligned next-generation sequencing data. For alignment, the Bowtie2 tool (https://github.com/BenLangmead/bowtie2) [34] was used, while FreeBayes [35] served as the SNP caller. However, even minor genomic variations can generate many variant calls across the genome. Thus, selecting a reliable variant caller for both SNVs and indels is critical. Further evaluation and refinement of the selected tool are essential to ensure maximum efficiency in data analysis. The study used four different aligners for analysis.

### Functional and comparative genomic annotation

The comparative genome analysis was conducted using the BV-BRC server, which integrates two PATRIC tools (Pathosystems Resource Integration Center) – the Protein Family Sorter and the Comparative Pathways Viewer – along with subsystems (https://www.bv-brc.org/app/ComparativeSystems) [36, 37]. These tools use two types of protein families: Protein local families (PLFams) for intragenus comparisons and PGFams for cross genus analyses [38]. The Protein Family Sorter tool employs clustering algorithms to examine the distribution patterns of protein families using three options: (1) identifying the core genome by selecting proteins present in all families, (2) analyzing the accessory genome by filtering proteins absent from all families, and (3) utilizing the default mixed/either option to define the pangenome.

### Gene prediction and functional annotation

The GeneMarkS-2 server developed by the Georgia Institute of Technology was used to process the assembled contig files that were produced in the earlier stages on the BV-BRC website (https://genemark.bme.gatech.edu/genemarks2.cgi). GeneMarkS-2 employs Hidden Markov Models (HMMs) and self-training algorithms to predict protein-coding genes in prokaryotic and eukaryotic genomes [39]. The EasyGene 1.2 server, developed at the Technical University of Denmark, generates a list of predicted genes given a sequence of prokaryotic DNA using HMMs (https://services.healthtech.dtu.dk/services/EasyGene-1.2/) [40]. Each prediction is given with a significance score (R-value) indicating the probability of being a noncoding open reading frame rather than a true gene. The predicted genes from different genotypes were subjected to BLASTN (Basic Local Alignment Search Tool for Nucleotides) and subjected to multiple sequence alignments (MSAs) using the ClustalW algorithm in BioEdit 7.2 software, developed by Tom Hall at Ibis Therapeutics, from different genotypes. Sections of DNA or protein sequences that have remained intact throughout evolution and include functional components such as structural motifs, non-coding regulatory domains, or protein-coding sections are referred to as conserved areas. By comparing sequences from various animals or people, these regions were found. The BioEdit 7.2 software’s “find conserved regions” function was used with the default settings of minimum length = 15 and maximum average entropy = 0.2 to identify the conserved sections in the 10 predicted genes. These criteria ensure that regions are recognized and meet the requirements for length and similarity for classification as described by Zhang *et al*. [41].

### Phylogenetic analysis of predicted genes

The phylogenetic analysis of the 10 predicted genes was conducted using MEGA11 software, developed by the Kumar Laboratory at Temple University, Clustal Omega, developed by the European Bioinformatics Institute (EBI), and the ClustalW tool. MEGA11 employs ML, neighbor-joining (NJ), and bayesian inference (BI) methods for phylogenetic tree reconstruction [42]. Clustal Omega and ClustalW utilize HMMs and guide trees for MSA before phylogenetic analysis [43]. However, the use of the ClustalW tool hindered the analysis. The type of nucleotide substitution and the selection of the best model, the General Time Reversible Model (GTR), were the subjects of the phylogenetic study.

### Statistical analysis

All statistical analyses were conducted using IBM Statistical Package for the Social Sciences Statistics for Windows, Version 21.0 (IBM Corp., Armonk, NY, USA). Descriptive statistics were used to summarize seroprevalence data, PCR results, and genomic characteristics of MAP isolates.

To determine the association between MAP infection rates and geographical sampling locations, a Chi-square (χ^2^) test was applied. The test assessed whether the distribution of MAP-positive cases varied significantly across different regions. A p < 0.05 was considered statistically significant, with a confidence interval of 95%.

For genome-wide comparisons, SNV and insertion/deletion (indel) frequencies were analyzed using standard bioinformatic pipelines. The statistical significance of phylogenetic clustering was evaluated using bootstrap analysis with 100 replicates in RAxML to ensure robustness.

Correlation analyses were performed where applicable to assess the relationship between MAP strain type (S-type vs. C-type) and clinical outcomes in camels. Fisher’s exact test was used for categorical data where sample sizes were small.

All results are presented as mean ± standard deviation or percentages, and statistical significance thresholds were set at α = 0.05.

## RESULTS

The total seroprevalence rate in the examined areas was 8.11%. Table 3 illustrates the percentage of positive serum samples in each region using ELISA. The Chi-square test showed that there was a significant correlation (alpha <0.05 and Chi-square = 34.47) between the infection rate and area of sample collection (p = 0.0010).

**Table 3:** Seroprevalence of JD in camels by ELISA in relation to sample area.

Region	Number of samples	Seroprevalence by ELISA	Positive (%)
Al Zaydiyah	47	3	6.38
Haradh	37	4	10.81
Alsaih	8	1	12.5
Salwa	31	1	3.22
Hofuf	16	2	12.5
Tamani	8	0	0
Al Udayd	8	0	0
Fadillah	10	0	0
Ahsa Market	36	0	0
Alghwaibah	20	5	25
Udhailiyah	22	1	4.54
Assarrar	79	4	5.06
Hanidh	10	2	20
Elwnan	13	5	38.46
Total	345	28	8.11

ELISA=Enzyme-linked immunosorbent assay, JD=Johne’s disease

**Figure 1:**
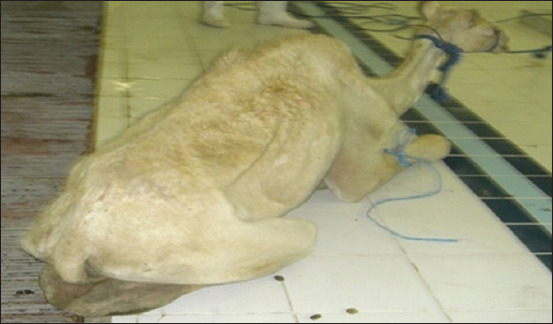
Highly emaciated camel with a large area of fur loss.

**Figure 2:**
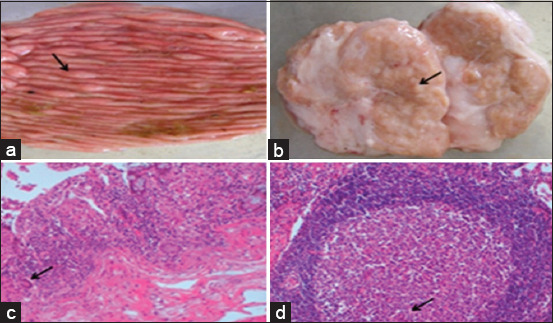
(a) Colon: Transverse corrugations of mucosal surface (arrow). (b) Mesenteric lymph node: Granular surface (arrow). (c) Colon: Macrophages, epithelioid, and few giant cells in the submucosa (arrow). Hematoxylin and Eosin (HE) bar = 20 μm. (d) Mesenteric lymph node: Hyperplastic activation with few numbers of giant cells in germinal centers (arrow). HE bar = 20 μm.

The characteristic clinical signs of JD were recorded during the clinical examination of the diseased camels. These signs include progressive emaciation (Figure 1), persistent diarrhea that did not respond to traditional antidiarrheal drugs, dehydration, and multiple areas of fur loss. Slaughtered camels showed gelatinous atrophy of the subcutaneous and visceral fats. The most noticeable gross lesions were intestinal wall thickening and corrugation. The mucosa was folded into transverse ridges that could not be reduced by stretching (Figure 2a). There were edema and congestion in the mesenteric and ileocecal lymph nodes (Figure 2b). Consistent microscopic findings in the ileum included fused villi. In most cases, the intestinal villi were short, blunt, and distorted. The mucosal surfaces of the ileum and colon indicate hyperactivation of the crypts of Lieberkuhn. The submucosa and lamina propria had significant infiltration of mononuclear cells, primarily macrophages (Figure 2c) as well as a few numbers of eosinophils everywhere. The most salient findings in the mesenteric and ileocecal lymph nodes were lymphocytic hyperplasia. The cortical lymphatic nodules and medullary cords were markedly hyperplastic. Multinucleated cells were noticed elsewhere (Figure 2d). Medullary sinus histiocytosis was obvious in most cases.

**Figure 3:**
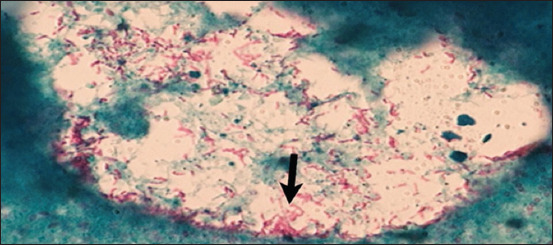
Acid fast bacilli of *Mycobacterium avium* subsp. *paratuberculosis* in rectal smear (100×).

**Figure 4:**
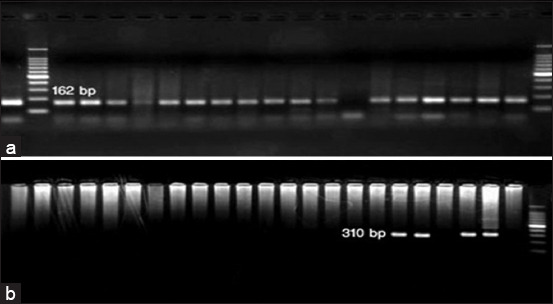
(a) *Mycobacterium avium* subsp. *paratuberculosis*-sheep strain at 162 bp; (b) *Mycobacterium avium* subsp. *paratuberculosis*-cattle strain at 310 bp.

Eight of the 68 samples showed typical acid–fast bacilli after staining with ZN stain (Figure 3). Among the 30 samples positive by PCR, 20 samples showed a clear band at 162 bp, indicating the MAP-S strain, and only 10 showed a clear band at 310 bp, indicating the MAP-C strain (Figures 4a and b). Table 4 showes the number of positive samples by ZN stain and PCR as well as the type of MAP relation to area of collection.

**Table 4:** Number of positive rectal smears by ZN staining and PCR.

Region	Number of samples	Number of positive samples by ZN stain	Genomic distribution		

			Number of positive PCR samples	S type	C type
Al Zaydiyah	8	2	4	2	2
Haradh	7	1	3	3	0
Alsaih	1	0	0	0	0
Salwa	11	2	7	4	3
Hofuf	7	1	7	5	2
Tamani	2	0	2	1	1
Al Udayd	4	1	4	3	1
Fadillah	1	0	0	0	0
Ahsa Market	10	0	1	0	1
Alghwaibah	3	0	0	0	0
Udhailiyah	3	0	0	0	0
Assarrar	1	0	0	0	0
Hanidh	1	0	0	0	0
Elwnan	9	1	2	2	0
Total	68	8	30	20	10

PCR=Polymerase chain reaction, ZN: Ziehl-Neelsen, S type=Sheep type, C type=Cattle type

A comprehensive genomic analysis of the MAPgenome was conducted. There have been reports of isolates of MAP JQ5 and JQ6 from Saudi Arabia. The genomic assemblies of the JQ5 and JQ6 strains revealed very identical features; 176 contig, 68.5% guanine-cytosine (GC) content, and 28 L50 count, which is referred to as the minimum number of contig whose length summation yields N50. However, there were minor differences in the genome lengths of 4735471 bp and 4697010 bp and in N50, which, at 50% of the genome, is acknowledged as the smallest sequence length, 54856 bp and 54634 bp for JQ5 and JQ6, respectively.

**Figure 5:**
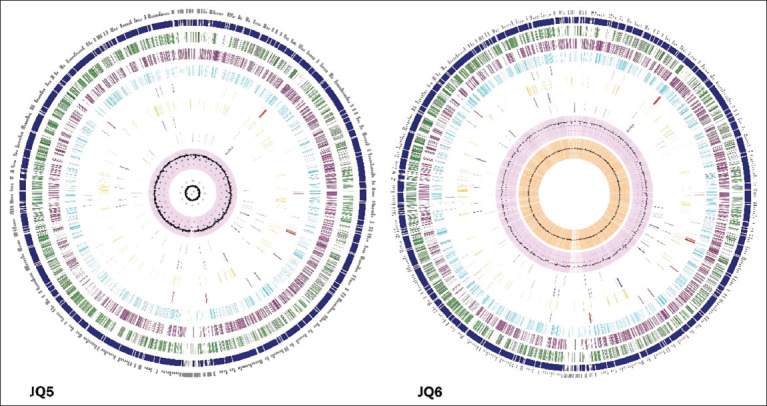
The circular genomes of the *Mycobacterium avium* subsp. *paratuberculosis* JQ5 and JQ6 strains are illustrated. The figure represents various genomic annotations, arranged from outside to inside, such as contig, coding DNA sequences (CDS) on the forward and reverse strands, transfer RNA, CDS of *antimicrobial resistance* genes, CDS of virulence factor, guanine–cytosine (GC) content, and GC skew.

An average of 4911 coding DNA sequences (CDS), 46 transfer RNA genes (tRNA), 3 ribosomal RNA genes (rRNA), 594 pseudogenes, 1595 putative proteins, and 3316 functional proteins were found in the MAP JQ5 strain genome annotation (Figure 5). An average of 1205 Enzyme Commission (EC) numbers, 1066 Gene Ontology (GO) numbers, and 959 proteins connected to Kyoto Encyclopedia of Genes and Genomes (KEGG) pathways in MAP are among these functional proteins. The PATRIC annotation identified 4792 PLFams and PGFams proteins (Table 5). In addition, the MAP JQ6 strain revealed 5098 CDS, 46 tRNA, 3 rRNA, 791 pseudogenes, 1695 hypothetical proteins, and 3403 functional proteins (Figure 5). Of these predicted functional proteins, 1256 had EC numbers, 1107 had GO, and 993 had KEGG pathway links. PLFams and PGFams proteins were 5024 in the PATRIC annotation additionally (Table 5).

**Table 5:** Genome assembly and annotation of MAP strains using the BV-BRC server.

Features	JQ5	JQ6
Genome assembly		
Contigs	176	176
Contigs L50	28	28
Contigs N50	54856 bp	54634 bp
GC content	68.5%	68.5%
Genome Length	4735471 bp	4697010 bp
Genomic features		
CDS	4911	5098
Pseudogenes	594	791
tRNA	46	46
rRNA	3	3
Protein features		
Hypothetical proteins	1595	1695
Functional proteins	3316	3403
Proteins have an EC number	1205	1256
Proteins contain GO	1066	1107
Proteins with a pathway	959	993
PLfam	4792	5024
PGfam	4792	5024

EC=Enzyme commission, GO=Gene ontology, PLfam=Protein local families, PGfam=Protein global families, tRNA=Transfer RNA, rRNA=Ribosomal RNA, CDS=Coding DNA sequences, BV-BRC=Bacterial and Viral Bioinformatics Resource Center, MAP=*Mycobacterium avium* subsp. *paratuberculosis*

Many of the identified genes in MAP share characteristics with widely recognized genes linked to drug targets, transporters, antibiotic resistance, and virulence factors, as indicated in (Table 6), while the AMR patterns of JQ5 and JQ6 are shown in (Table 7).

**Table 6:** Candidate genes of the MAP JQ5 and JQ6 strains.

Categories	Source	JQ5	JQ6
	
		Genes	Genes
Transporter	TCDB	43	48
Human homolog	Human	39	44
Antibiotic resistance	CARD	13	13
	PATRIC	43	44
Virulence factor	PATRIC_VF	180	179
	VFDB	17	19
	Victors	113	113
Drug target	Drug target	44	44
	TTD	22	19

MAP=*Mycobacterium avium* subsp. *paratuberculosis*, TCDB=Transporter Classification Database, CARD=Comprehensive Antibiotic Resistance Database, VFDB=Virulence factor database, TTD=Therapeutic Targets Database

**Table 7:** AMR genes of the two MAP strains.

AMR mechanism	Genes
Antibiotic activation enzymes	*KatG*
Antibiotic targets in susceptible species	*Alr, dxr, EF-G, EF-Tu, EmbA, EmbB, EmbC, folA, Dfr, folP, gyrA, gyrB, inhA, fabI, Iso-tRNA, kasA, MurA, rho, rpoB, rpoC, S10p, S12p*
Antibiotic target protein	*MfpA*
Antibiotic target replacement protein	*FabG, HtdX*
Efflux pump resistance-conferring antibiotic resistance	*MmpL5, Rv2994*
Protein-altering cell wall charges confer antibiotic resistance	*PgsA*
Regulator modulating expression of antibiotic resistance gene expression	*EmbR, EthR, MtrA, MtrB,* and*OxyR*

MAP=*Mycobacterium avium* subsp. *paratuberculosis*, AMR=Antimicrobial resistance

**Figure 6:**
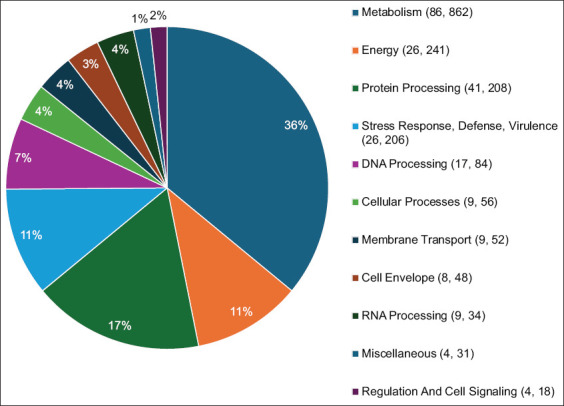
Distribution of subsystem categories for *Mycobacterium avium* subsp. *paratuberculosis* JQ5, which are organized through this pattern Subsystem Counts (Subsystems, Genes).

**Figure 7:**
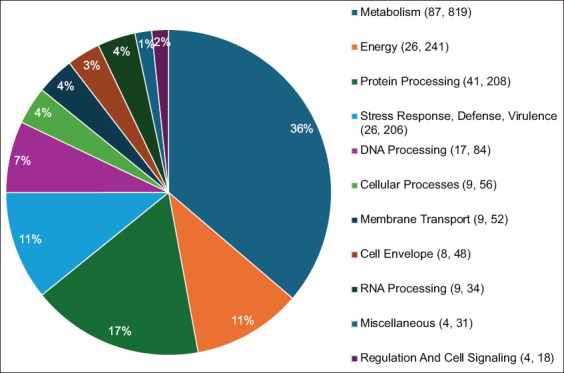
Distribution of subsystem categories for *Mycobacterium avium* subsp. *paratuberculosis* JQ6 organized through this pattern Subsystem Counts (Subsystems, Genes).

A group of proteins called subsystems cooperate to perform specific biological functions or structural complexes. The features and coverage of each subsystem are summarized in a pie chart (Figures 6 and 7). The data were arranged in accordance with this pattern of Subsystem Counts (Subsystems, Genes), indicating that the metabolism of the MAP JQ5 strain is a distinct biological process with 86 subsystems under the control of 862 genes (Figures 6 and 7).

**Figure 8:**
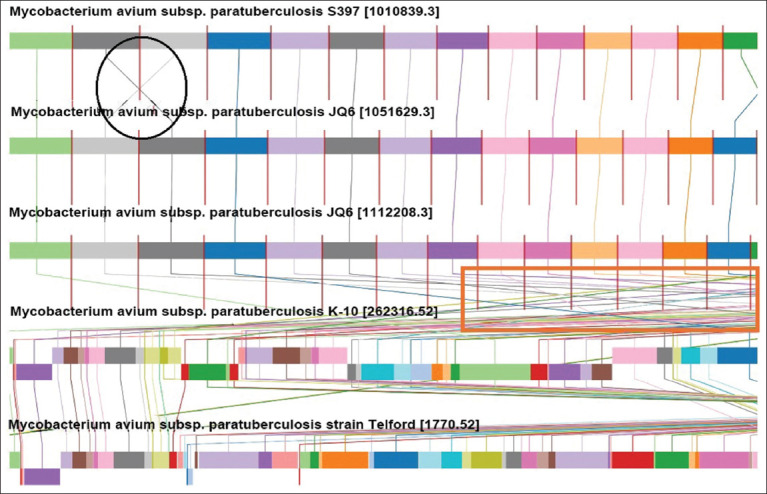
The multiple sequence alignment of *Mycobacterium avium* subsp. *paratuberculosis* is illustrated. The black circle represents the inversion region between two genomes, whereas the colored lines between genomes in the red box represent rearrangements of genomic sequences.

The MAP strains S397 (S-strain type III), Telford (S-strain type I), and K-10 (C-strain type II) were the reference genomes for the MSAs of the MAP genomes of JQ5 and JQ6, as illustrated in (Figure 8) using the progressive Mauve alignment program (Aaron Darling and colleagues at the University of Wisconsin-Madison) and every genome has its name listed horizontally on a black horizontal center line. The homologous segments are depicted by colored blocks, and the s-shaped rearrangements or inversions between the genomes are indicated by colored lines. Blocks above the center line denote alignment with the original genomic sequence in the forward direction; blocks below the line denote alignment with the original genomic sequence in the reverse complement (inverse) direction.

**Figure 9:**
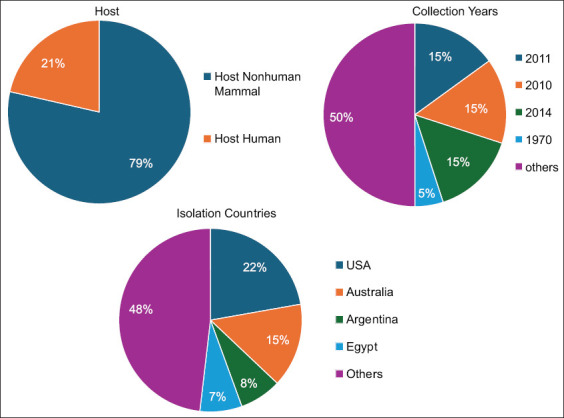
The distribution of the whole genomes used in the phylogenetic tree.

We constructed a whole-genome phylogenetic tree using 35 genomes isolated from diverse hosts, geographic regions, and time periods, including 26 MAP genomes and 9 outgroup genomes (Figure 9). The tree was generated based on protein and gene sequences from 1000 conserved single-copy genes. To ensure robustness, 100 rapid bootstrap replicates were performed, and most branches exhibited strong bootstrap support, indicating reliable phylogenetic relationships.

**Figure 10:**
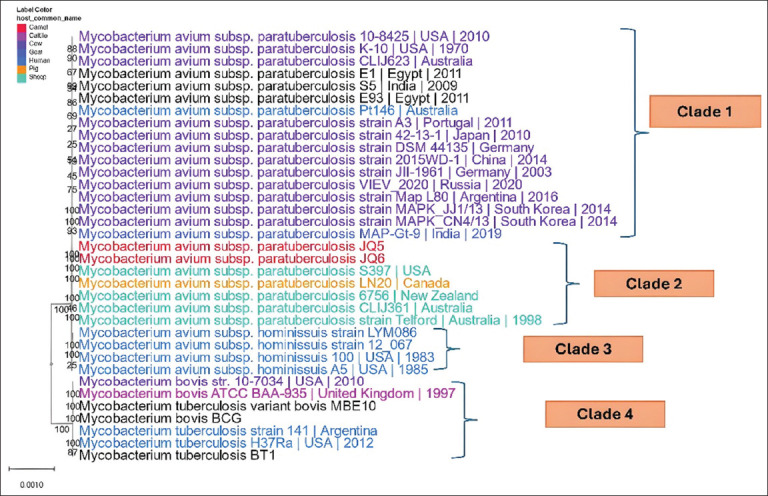
The whole genome phylogeny (26 genomes) of *Mycobacterium avium* subsp. *paratuberculosis* from different hosts. The tree was built on the protein and gene sequences of the 1000 genes performed at the PATRIC server. The branch support values were obtained using 100 bootstrap values, and the length of a branch was relative to the total number of changes at each site.

The phylogenetic analysis revealed four distinct clades. Clade 1 predominantly comprises C-type II MAP strains that infect cattle, with a few goat-associated strains. Clade 2 clusters S-type I and III strains, primarily found in camels, sheep, and pigs. Clade 3 includes human-associated *M. avium* subsp. *hominissuis* strains, suggesting potential zoonotic implications. The inclusion of an outgroup (*Mycobacterium* *bovis* and *Mycobacterium* *tuberculosis*) provided a clearer evolutionary framework, reinforcing the divergence between MAP strains and other members of the *Mycobacterium* genus (Figure 10).

The Bowtie2 tool was used to identify 9285 SNPs in all clades 2 genomes relative to the reference genome MAP JQ5. These variants were annotated using SnpEff (Pablo Cingolani at Queensland University of Technology) [44]. SnpEff employs a rule-based annotation algorithm to match variants to genome annotations and predict their functional impact based on predefined biological rules. The analysis classified the variants into 6175 insertions, 842 synonymous variants, and 463 deletions. In addition, 2898 frameshift mutations, 1971 intergenic region variants, and 3109 missense variants were predicted. The impact of these variants included 2018 modifiers, 3461 moderate-impact variants, 819 low-impact variants, and 3008 high-impact variants.

The pangenome represents the complete set of shared and unique genetic material within a species, encompassing the combined genetic content of all sampled genomes. This analysis provides insights into the composition, diversity, and evolutionary dynamics of gene content within a population. The core genome comprises protein families that are conserved across all analyzed genomes, whereas the accessory genome includes those present only in specific subsets.

For MAP across 26 genomes, pangenome analysis identified 1984 core genes, no accessory genes, and a total pangenome size of 3977 genes. These findings suggest a high degree of genetic similarity among the MAP strains, with minimal gene variation and a largely conserved genome structure.

The GeneMarkS-2 server was used to predict MAP JQ5 strain. This analysis revealed 3438 predicted genes for MAP. As a result, we submitted the query genes that were most similar to the database sequence (10) and obtained accession numbers for these genes. Each gene had a variable average entropy (Hx), segment length, and number of conserved portions when using BIOEDIT for MSA (Table 8).

**Table 8:** The predicted genes with accession numbers and conserved regions of submitted genes for MAP using BioEdit.

Isolate	Gene	Length (bp)	Accession Numbers	Number of conserved regions	Average entropy (Hx)	Function	References
KSA	*Mce2A*	735	LC843448	16	0.0000	Virulence factors enhance bacterial entry into host cells and contribute to immune evasion by modulating host responses.	[12]
	*mce4A*	1223	LC843449	24	0.0000	Virulence factor, which is a Mce family member, is involved in lipid metabolism. It facilitates the survival of MAPs within macrophages using host lipids.	[12,13]
	*gyrA*	2128	LC843450	29	0.0000	Antibiotic resistance. They play critical roles in bacterial growth and survival. The subunit of DNA gyrase is essential for DNA replication and supercoiling.	[14,15]
	*gyrB*	2067	LC843451	33	0.0000		
	*sodA*	634	LC843452	14	0.0000	Helps MAPs survive the oxidative burst of host immune cells, enhancing their persistence in the host.	[16]
	*mbtG*	1113	LC843453	18	0.0000	Involved in mycobacterial cell wall biosynthesis.	[17]
	*PknD*	769	LC843454	9	0.0000	A serine/threonine protein kinase is involved in signaling pathways that contribute to virulence.	[18]
	*sigE*	768	LC843455	11	0.0000	The sigma factor contributes to adapting to the host environment and modulating immune responses during infection.	[12,18]
	*Pks12*	1369	LC843456	4	0.0000	Virulence factors involved in the biosynthesis of complex lipids.	[18]
	*pknG*	1290	LC843457	23	0.0000	Virulence factors and drug target candidate. It plays different roles in MAP pathogenicity: aggregation, clumping, and biofilm formation, immune invasion, and establishment.	[19]

MAP=*Mycobacterium avium* subsp. *paratuberculosis*

The GTR model was used to estimate substitution patterns and rates. The rates for various transitional substitutes are displayed in bold, whereas the rates for transversion substitutions are displayed in italics (Table 9). The relative values of instantaneous r should be considered when evaluating them. For simplicity, the sum of r values was 100. The nucleotide frequencies were A = 17.84%, T/U = 15.32%, C = 33.80%, and G = 33.05%. The estimated transition/transversion bias (R) was 0.72.

**Table 9:** Maximum likelihood estimation of substitution matrix

Nucleotide	A	T/U	C	G
A	-	*8.08*	*6.47*	**14.64**
T/U	*9.41*	-	**14.97**	*6.06*
C	*3.42*	**6.79**	-	*9.61*
G	**7.90**	*2.81*	*9.83*	-

*The rates for various transitional substitutes are displayed in bold, whereas the rates for transversion substitutions are displayed in italics

**Figure 11:**
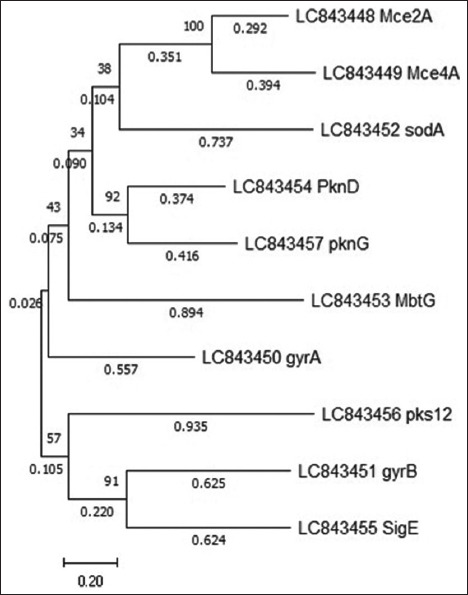
The evolutionary history was inferred using the Maximum Likelihood method and General Time Reversible Model. The initial tree(s) for the heuristic search were obtained automatically by applying the neighbor-join and BioNJ algorithms to a matrix of pairwise distances estimated using the Maximum Composite Likelihood approach and then selecting the topology with the superior log likelihood value. The tree is drawn to scale, with branch lengths measured as the number of substitutions per site (next to the branches). The analysis involved 10 nucleotide sequences. There were 2263 positions in the final dataset.

The Maximum Likelihood approach was applied to assess the 10 predicted genes using MEGA11 software. The GTR model was determined to be the most effective model for characterizing the substitution pattern based on the lowest Bayesian Information Criterion scores. The tree with the highest log likelihood (−15198.57) is shown in Figure 11.

## DISCUSSION

The total seroprevalence rate of antibodies tested by ELISA in the examined areas was 8.11%, which is in accordance with many early studies in Kingdom of Saudia Arabia, especially the Eastern region [16, 45], Oman [46], and Egypt [17]. The recent seroprevalence of *Mycobacterium paratuberculosis* (MPT) in the eastern region recorded the same range between 7% and 10% [47–49]. In the present study, there was a significant correlation (alpha <0.05 and Chi-square = 34.47) between the infection rate and area of sample collection at p = 0.0010, although more investigation is needed with a larger statistical sample size. The investigation documented severe emaciation and persistent diarrhea, as observed clinical symptoms, as described by Tigani-Asil *et al*. [50] and Salem *et al*. [51]. The clinical signs vary according to the clinical form of the disease [52]. Moreover, the disease was observed in a 2-year-old patient, which is consistent with Spickler [52] and Gameel *et al*. [53]. According to Ivanov and Skalinskii [54], the disease is more common in camels between the ages of two and five, and it usually results in death between 2 and 20 weeks of the disease’s onset. The most noticeable gross lesions in the current investigation were intestinal wall thickening and corrugation, which were associated with persistent purulent enteritis. These results agree with those of Spickler [52] and Tharwat *et al*. [55]. Many other authors and textbooks have noted these pathognomonic lesions of paratuberculosis (PTB). Chronic, slowly proliferative, or granulomatous enteritis causes the mucosa to thicken diffusely into transverse folds, which is the pathological appearance of PTB clinical cases in genuine hosts [52, 55, 56]. The histopathology results described in this study agree with those of previous studies by Spickler [52], Tharwat *et al*. [55] and Hailat *et al*. [56] as the sections of the mesenteric lymph nodes showed a broad lepromatous reaction devoid of caseation and calcification, with a proliferating population of macrophages and epithelioid cells combined with lymphocytes and other mononuclear cells. Long-term infections may lower immunity and cause a variety of diseases, which could account for the intestinal damage observed in the intestine and other organs of camels infected with paratuberculosis [57]. Ziehl–Neelsen stain was used in the current study to confirm the traditional diagnosis of PTB in camels. The staining smears from the mesenteric lymph nodes and ileocecal mucosa revealed many densely packed, acid–fast MAP phagosomes lodged inside the cytoplasm of macrophages and epithelioid cells, which is in accordance with previous studies by Elmoslemany *et al*. [47], Tigani-Asil *et al*. [50] and Salem *et al*. [51]. In the present study, specific DMC-PCR was used for the detection and differentiation of both strains of MAP to determine the source of infection in studying the epidemiology of the disease. Several studies by Clark *et al*. [27], Garrido *et al*. [58] and Ikiz *et al*. [59] have detected MAP-infected animals using PCR. In this study, the results of PCR recorded that the S-strain of MPT is more prevalent in camels, which is in accordance with Elsohaby *et al*. [49]; such interpretation is documented epidemiologically by nature of grassing camels with sheep more than that with cattle [18].

Identifying the specific genes could provide crucial insights into MAP’s pathogenesis and pave the way for developing a vaccine for paratuberculosis through targeted gene knockout. Annotating and comparing the genomes of different MAP strains are crucial for uncovering their genetic characteristics and identifying variations, such as single-nucleotide polymorphisms (SNPs), which help in validating vaccine strains. In this study, the annotated MAP strains exhibited remarkable similarities in various genetic features, consistent with previous findings by Ghosh *et al*. [18].

Identifying the specific genes could provide crucial insights into MAP’s pathogenesis and pave the way for developing a vaccine for paratuberculosis through targeted gene knockout. Annotating and comparing the genomes of different MAP strains are crucial for uncovering their genetic characteristics and identifying variations, such as SNPs, which help in validating vaccine strains. In this study, the annotated MAP strains exhibited remarkable similarities in various genetic features, consistent with previous findings by Ghosh *et al*. [18].

In this study, 34 *AMR* genes were identified in MAP using the PATRIC server. The stealthy nature of MAP is due to its various AMR mechanisms, which include antibiotic-activating enzymes, species-specific target proteins, efflux pumps, target protection and replacement proteins, proteins that alter cell wall charge, and regulators that control resistance gene expression. The *katG* gene encodes catalase-peroxidase, which activates isoniazid, a key drug used against mycobacterial infections, leading to resistance when mutated [60]. One key enzyme, DNA gyrase (gyrA and gyrB), plays a critical role in the supercoiling of DNA, and targeting this enzyme with quinolones or fluoroquinolones leads to the rapid killing of MAP [61]. On the other hand, rifampicin, which inhibits DNA-dependent RNA polymerase, consists of four key subunits from A to D (rpoA-D). Resistance to rifampicin typically arises from a mutation in the *rpoB* gene, specifically at the 81 base pair [62, 63]. The *inhA* gene encodes an enoyl-acyl carrier protein reductase that is involved in mycolic acid synthesis and contributes to resistance to isoniazid and ethionamide [64].

In addition, genes such as *S10p*, *S12p*, *Alr*, *Ddl*, *EF-G*, *inhA*, *Dfr*, *kasA*, *rho*, *EF-Tu*, *Iso-tRNA*, *MurA*, and *fabI* are involved in a variety of important cellular functions and pathways in MAP. MurA plays a role in peptidoglycan biosynthesis, making it a target for fosfomycin, while fabI contributes to resistance against triclosan by affecting fatty acid synthesis [65]. Dfr and folP encode enzymes involved in folate biosynthesis, conferring resistance to trimethoprim and sulfonamides [66]. MfpA protein acts as a DNA mimic to protect DNA gyrase against fluoroquinolone inhibition, whereas target replacement proteins, such as FabG and HtdX, modify essential enzyme targets to maintain bacterial viability in the presence of antibiotics [61, 67].

Furthermore, *EmbA*, *EmbB*, and *EmbC* are associated with ethambutol resistance by modifying arabinogalactan biosynthesis [68–70]. *Alr* and *dxr* genes contribute to resistance mechanisms affecting alanine racemase inhibitors and fosmidomycin, respectively [71]. Ribosomal proteins such as S10p and S12p are involved in maintaining translational fidelity but can develop mutations leading to resistance to aminoglycosides [72]. EF-G and EF-Tu regulate translation elongation and have been implicated in resistance to fusidic acid [73]. Iso-tRNA modification affects susceptibility to antibiotics that target protein synthesis [74]. The antibiotic target protection protein MfpA shields bacterial DNA gyrase from fluoroquinolones, thereby reducing the effectiveness of the drugs [67, 75]. Meanwhile, the target replacement proteins FabG and HtdX modify or replace antibiotic targets within the bacteria, allowing them to perform essential processes despite the presence of these drugs [76].

Moreover, Efflux pumps such as MmpL5 and Rv2994 actively remove antimicrobial agents from bacterial cells, reducing intracellular drug accumulation and conferring multidrug resistance. This process lowers the concentration of drugs inside the cell, thereby making them less effective [49]. Regulatory genes, including *EmbR*, *EthR*, *MtrA*, *MtrB*, and *OxyR*, modulate the expression of multiple *AMR*-related genes, enhancing MAP’s resistance profile. Together, these genes and their associated mechanisms significantly contribute to MAP’s ability to survive in the presence of antimicrobial treatments, complicating therapeutic approaches for controlling paratuberculosis. In MAP, the transcription factor OxyR helps the bacteria battle oxidative stress caused by reactive oxygen species (ROS) generated by the host’s immune system. OxyR regulates the expression of various cellular components involved in antioxidant defense, activating genes such as catalase-peroxidase, superoxide dismutase, and other detoxification-related genes. These mechanisms help protect bacteria from damage by ROS [77].

PgsA also reduces the effectiveness of positively charged antibiotics, such as aminoglycosides and certain peptides, by preventing them from binding to the bacterial cell surface and entering the cell. This adaptation enables MAP to survive in environments with high antibiotic concentrations, allowing it to persist inside host macrophages and resist treatments for paratuberculosis [78, 79].

In this study, MSA was conducted and revealed that the JO5 and JO6 strains were almost aligned with the reference genome MAP_*S397* (S-strain type III) but different from MAP_K10 (C-strain type II). This was confirmed by whole-genome phylogeny analysis. It was classified into four clades: C-strain, S-strain, Human strain, and outgroup. The isolates of this study were related to S. The S-strain included different hosts – sheep, camel, and pig – which suggests that it can easily cross-species transmit, pointing to a wider ecological role. In contrast, the C-strain, which is mainly found in cattle, exhibits a more specific host preference. This difference emphasizes the importance of customized monitoring and control strategies for each strain to manage its spread effectively.

Although this study did not directly analyze horizontal gene transfer (HGT), the observed phylogenetic relationships suggest potential genetic exchanges that may facilitate host adaptation. Previous studies by Ivanov and Skalinskii [54], Tharwat *et al*. [55], Hailat *et al*. [56] have identified mobile genetic elements such as insertion sequences and integrative conjugative elements, which could contribute to genetic variability and interspecies transmission. The close genetic relationship between strains from different hosts raises the possibility of shared genetic adaptations, potentially mediated by HGT, that enhance MAP’s ability to infect multiple species. Although our phylogenetic analysis alone cannot confirm such events, the clustering patterns suggest a need for further comparative genomic studies focusing on recombination hotspots and mobile genetic elements to clarify the role of HGT in MAP evolution.

In addition, several genes were predicted to play a role in MAP virulence and antibiotic resistance. For instance, the *mce2A* and *mce4A* genes, which are part of the multidrug resistance (mce) operons, help the bacterium survive in tough environments, potentially supporting its persistence within host macrophages [80]. The *sodA* gene encodes superoxide dismutase, an enzyme that helps MAP cope with oxidative stress, improving its survival during immune attacks [81]. In addition, the *mbtG* gene is involved in the production of mycobactin, a siderophore that is essential for iron acquisition, which is crucial for bacterial growth and virulence [82]. The *PknD* and *PknG* genes are members of the Ser/Thr protein kinase family and contribute to signaling pathways that regulate various cellular processes, including antibiotic resistance. The sigma factor sigE plays a key role in regulating stress response genes, whereas pks12 is linked to the production of polyketide, which may support the bacterium’s virulence [76]. Together, these genes increase MAP adaptability and resilience, making the treatment and control of paratuberculosis in livestock particularly challenging.

To strengthen our understanding of these virulence genes, a comparative analysis with previously published datasets from MAP isolates from different hosts is valuable. Studies have reported variations in the expression and expression of virulence genes among MAP strains isolated from cattle, sheep, goats, and wildlife. For example, differences in the mce operon composition and regulation have been noted between bovine and ovine MAP isolates, potentially influencing host specificity and immune evasion strategies [56, 59, 64, 68]. In addition, variations in sigE expression levels among host-adapted MAP strains may contribute to differential stress tolerance and persistence. By integrating such comparative genomic data, we can better understand host-specific adaptations and the evolutionary pressures shaping MAP virulence. Further research analyzing these genes across multiple host species will provide insights into MAP’s pathogenic mechanisms of MAPs and could help refine disease control strategies in livestock.

The high number of mutations identified in the SNP analysis of clade 2 genomes compared with the reference genome MAP JQ5 suggests significant genetic diversity and explains cross-species transmission between camels, sheep, and pigs within clade 2. This diversity may result from evolutionary divergence, adaptation to varying environmental conditions, or selective pressure exerted by host immune systems. The presence of 2898 frameshift mutations further indicated potential disruptions of gene function, which could have substantial phenotypic consequences. In addition, the identification of 1971 variants in intergenic regions indicates possible alterations in regulatory elements, potentially affecting gene expression patterns. High-impact variants are particularly likely to have profound effects on protein function and overall pathogenicity. The observed genetic variation is consistent with the known capacity of *Mycobacterium* species to evolve rapidly in response to environmental and host-related pressures. These findings highlight the importance of understanding genetic diversity and mutation patterns in pathogen evolution, as they provide insights into the mechanisms of adaptation and potential targets for intervention. Further research could focus on elucidating the specific roles of these mutations in the biology and virulence of clade 2 *Mycobacterium* strains that target camel, sheep, and pigs.

The pan-genome analysis of the 26 MAP genomes suggested a highly conserved genome structure among the analyzed strains, indicating minimal gene gain or loss events. In contrast, SNP analysis provides further insights into the genetic diversity within these core genes, revealing that MAP primarily evolves through point mutations rather than HGT or major structural variations. The absence of accessory genes suggests that genetic adaptation in MAP strains is not driven by the acquisition of novel genes but rather by mutations within the conserved genetic framework. The high conservation of core genes may have implications for vaccine and drug development because it highlights potential targets that remain stable across diverse MAP populations. However, the observed SNP variations within the core genome may still contribute to functional differences among strains, influencing virulence, host adaptation, and AMR. Further comparative studies incorporating transcriptomic and functional assays could help clarify the impact of these genetic variations on MAP pathogenesis and host specificity.

Identifying these specific genes involved in MAP pathogenesis could guide vaccine design, particularly by targeting conserved antigens or knocking out virulence factors to develop an attenuated vaccine. Furthermore, the identification of genetic markers may support the development of a DIVA-compatible vaccine, allowing differentiation between infected and vaccinated animals.

## CONCLUSION

This study provides a comprehensive analysis of MAP infections in camels in the Eastern region of Saudi Arabia, integrating clinical, pathological, molecular, and genomic approaches. The results confirmed the presence of MAP infection in camels, with a seroprevalence rate of 8.11% detected through ELISA. Clinical examination of infected camels revealed progressive emaciation and chronic diarrhea, while postmortem findings indicated intestinal wall thickening and mesenteric lymph node congestion. Histopathological analysis showed fused villi, hyperactivation of crypts of Lieberkühn, and mononuclear cell infiltration, confirming the presence of JD-related lesions.

Molecular characterization identified higher prevalence of the MAP-S strain (162 bp) compared to the MAP-C strain (310 bp) through PCR, suggesting potential transmission from infected sheep populations. Whole-genome sequencing and bioinformatic analysis revealed that MAP strains in camels exhibit a high degree of genetic similarity to sheep strains, indicating possible cross-species transmission. The genomic annotation identified 34 *AMR* genes and 10 virulence-associated genes, highlighting the pathogen’s adaptation to host environments and its potential resistance to commonly used antimicrobials. Phylogenetic analysis clustered camel-derived MAP strains into four distinct clades, further supporting genetic divergence among host species.

This study integrates clinical, serological, molecular, and genomic techniques, providing a holistic understanding of MAP infections in camels. It is one of the first studies to conduct whole-genome sequencing and phylogenetic analysis of MAP isolates from camels in Saudi Arabia, contributing novel insights into strain diversity and transmission dynamics. The identification of *AMR* genes provides valuable information for developing targeted control strategies, especially considering the challenges of treating MAP infections.

Although 345 samples were analyzed, a larger dataset across multiple regions would enhance the generalizability of findings. While the genomic analysis suggests sheep-to-camel transmission, direct epidemiological tracking of transmission events was not performed. The study highlights the need for a camel-specific MAP vaccine, but further experimental studies are required to identify immunogenic targets.

Future research should conduct longitudinal studies across a broader geographic range to better understand the epidemiology and risk factors associated with MAP transmission. The identification of virulence genes and AMR markers provides a foundation for developing vaccines and antimicrobial treatment strategies tailored for camels. Further comparative genomic studies with MAP strains from different livestock species could elucidate host-specific adaptations and potential zoonotic risks. The findings call for enhanced MAP surveillance programs in camel herds to mitigate economic losses and potential public health risks.

This study contributes critical genomic and epidemiological insights into MAP infections in camels, emphasizing the urgent need for effective control measures, improved diagnostics, and vaccine development. By bridging the knowledge gap in MAP strain diversity, AMR, and cross-species transmission, these findings lay the groundwork for enhancing paratuberculosis control strategies in the Middle East and globally.

## AUTHORS’ CONTRIBUTIONS

MS: Conceptualized the study and revised the manuscript; AA: Conceptualized the study and edited and revised the manuscript. FH: Investigation and data collection; AE: Data analysis and edited and revised the manuscript. MEH and AE: Bioinformatic and revised the manuscript. KAS: Investigation and samples collection. NBA: Investigation and data analysis. AA: Data analysis and revised the manuscript. All authors have read and approved the final manuscript.
